# Ceramides improve cardiovascular risk prediction beyond low-density lipoprotein cholesterol

**DOI:** 10.1093/ehjopen/oeae001

**Published:** 2024-01-08

**Authors:** Andreas Leiherer, Axel Muendlein, Christoph H Saely, Reijo Laaksonen, Peter Fraunberger, Heinz Drexel

**Affiliations:** Vorarlberg Institute for Vascular Investigation and Treatment (VIVIT), Carinagasse 47, A-6800 Feldkirch, Austria; Private University of the Principality of Liechtenstein, Dorfstrasse 24, FL-9495 Triesen, Liechtenstein; Medical Central Laboratories, Carinagasse 41, A-6800 Feldkirch, Austria; Vorarlberg Institute for Vascular Investigation and Treatment (VIVIT), Carinagasse 47, A-6800 Feldkirch, Austria; Private University of the Principality of Liechtenstein, Dorfstrasse 24, FL-9495 Triesen, Liechtenstein; Vorarlberg Institute for Vascular Investigation and Treatment (VIVIT), Carinagasse 47, A-6800 Feldkirch, Austria; Private University of the Principality of Liechtenstein, Dorfstrasse 24, FL-9495 Triesen, Liechtenstein; Department of Internal Medicine III, Academic Teaching Hospital Feldkirch, Carinagasse 47, A-6800 Feldkirch, Austria; Finnish Cardiovascular Research Center, University of Tampere, FI-33014 Tampere, Finland; Zora Biosciences, FI-02150 Espoo, Finland; Private University of the Principality of Liechtenstein, Dorfstrasse 24, FL-9495 Triesen, Liechtenstein; Medical Central Laboratories, Carinagasse 41, A-6800 Feldkirch, Austria; Vorarlberg Institute for Vascular Investigation and Treatment (VIVIT), Carinagasse 47, A-6800 Feldkirch, Austria; Private University of the Principality of Liechtenstein, Dorfstrasse 24, FL-9495 Triesen, Liechtenstein; Vorarlberger Landeskrankenhausbetriebsgesellschaft, Academic Teaching Hospital Feldkirch, Carinagasse 47, A-6800 Feldkirch, Austria; Drexel University College of Medicine, Philadelphia, PA 19129, USA

**Keywords:** LDL, Cholesterol, Ceramide, Phosphatidylcholine, CERT, Cardiovascular risk prediction

## Abstract

**Aims:**

Low-density lipoprotein cholesterol (LDL-C) is the best documented cardiovascular risk predictor and at the same time serves as a target for lipid-lowering therapy. However, the power of LDL-C to predict risk is biased by advanced age, comorbidities, and medical treatment, all known to impact cholesterol levels. Consequently, such biased patient cohorts often feature a U-shaped or inverse association between LDL-C and cardiovascular or overall mortality. It is not clear whether these constraints for risk prediction may likewise apply to other lipid risk markers in particular to ceramides and phosphatidylcholines.

**Methods and results:**

In this observational cohort study, we recorded cardiovascular mortality in 1195 patients over a period of up to 16 years, comprising a total of 12 262 patient-years. The median age of patients at baseline was 67 years. All participants were either consecutively referred to elective coronary angiography or diagnosed with peripheral artery disease, indicating a high cardiovascular risk. At baseline, 51% of the patients were under statin therapy. We found a U-shaped association between LDL-C and cardiovascular mortality with a trough level of around 150 mg/dL of LDL-C. Cox regression analyses revealed that LDL-C and other cholesterol species failed to predict cardiovascular risk. In contrast, no U-shaped but linear association was found for ceramide- and phosphatidylcholine-containing markers and these markers were able to significantly predict the cardiovascular risk even after multivariate adjustment.

**Conclusion:**

We thus suggest that ceramides- and phosphatidylcholine-based predictors rather than LDL-C may be used for a more accurate cardiovascular risk prediction in high-risk patients.

## Graphical Abstract

**Graphical Abstract:**
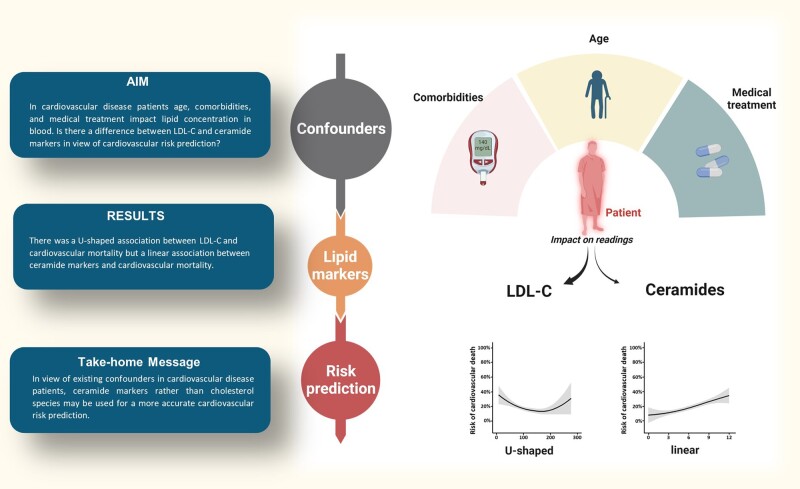

## Introduction

Numerous clinical and genetic studies have consistently demonstrated that low-density lipoprotein cholesterol (LDL-C) is the cause of atherosclerotic cardiovascular disease (ASCVD).^1^ Likewise, a range of clinical trials has unequivocally confirmed that reducing LDL-C levels lowers the risk of cardiovascular events and mortality.^1–4^ As a result, LDL-C has emerged as the most widely used marker for predicting cardiovascular risk and guiding treatment approaches in clinical practice.^1^

On the other hand, medical treatment, advanced age, and certain diseases (e.g. cachexia as seen in congestive heart failure, end-stage liver disease, or chronic obstructive pulmonary disease) are known to decrease cholesterol levels in particular LDL-C.^5–9^ Notably, a recent collaborative analysis of participants from the PROMINENT, REDUCE-IT, and STRENGTH trials, who are at high risk of atherosclerotic disease and receiving contemporary statins, found no association between LDL-C and major adverse cardiovascular events (MACE).^10^ Furthermore, several other studies have reported the absence^11–13^ or even an inverse association between LDL-C and cardiovascular risk^11,14–16^ as well as mortality,^9,12,17–22^ with many demonstrating a U-shaped risk curve and a vertex of around 150 mg/dL LDL-C.^9,20–22^ As a consequence, it has recently been suggested that such a relatively high value is not necessarily hazardous in itself.^20^ Moreover, LDL-C readings below this threshold may appear to confer a higher cardiovascular risk, indicating an inverse risk association. Hence, in patients characterized by advanced age, comorbidities, and undergoing lipid-lowering treatment, a poor prognosis with low LDL-C might be anticipated. While it has been recently explained that there is no real LDL-C paradox, because LDL-C is also a marker of overall frailty reflecting morbidity and biological ageing,^9^ LDL-C's value as a risk predictor in certain populations is quite limited. This limitation is also reflected in the recent recommendations of the consensus panel of the European Atherosclerosis Society and the European Federation of Clinical Chemistry and Laboratory Medicine, which has advocated for the measurement of advanced lipid profiles beyond LDL-C.^23^

The new lipid markers ceramides and phosphatidylcholines, when combined in a risk prediction score, have demonstrated remarkable potency as risk predictors, surpassing prediction based on cholesterol.^24^

Given the observed inverse or U-shaped association between LDL-C and mortality in certain study populations, particularly among those with advanced age, comorbidities, and undergoing statin treatment, our present study focused on a real-world population of hospitalized patients with a high cardiovascular risk. Considering the previously described constraints for LDL-C as a predictor of cardiovascular risk, we aimed to (i) analyse the role of LDL-C in our study population and (ii) investigate whether these limitations may likewise extend to other lipid markers, specifically ceramides and phosphatidylcholines, and their role as cardiovascular risk predictors.

## Methods

### Study subjects

This study comprised 1195 Austrian residents of Caucasian origin with a median age of 67 years [interquartile range (IQR): 58–74 years]. All participants were patients in the hospital's cardiology unit, with a presumed high cardiovascular disease risk. They were either referred to coronary angiography (CAG, *n* = 915) for the evaluation of established or suspected stable coronary artery disease using the Judkins technique or were diagnosed to have peripheral artery disease (PAD, *n* = 280) according to the primary manifestation of ischaemic pain in the lower extremities and Ankle-Brachial Index-Doppler ultrasound. Patients with acute coronary syndrome were not included. Prospective follow-up was conducted for up to 16 years, and follow-up data were available for 1185 out of 1195 patients. Outcome data, in particular time and causes of death or other events, were obtained from hospital records and were collected annually from a national survey (Statistik Austria, Vienna, Austria). For the assessment of non-fatal endpoints, we conducted standardized interviews biannually. The primary study endpoint was cardiovascular death (fatal myocardial infarction, sudden cardiac death, mortality from congestive heart failure due to coronary artery disease). The secondary endpoints included all-cause death and MACE (cardiovascular death, fatal ischaemic stroke, non-fatal myocardial infarction, and non-fatal ischaemic stroke).

### Clinical and laboratory analyses

Basic clinical measurements and laboratory analyses were performed as described in detail elsewhere.^25^ Type 2 diabetes mellitus (T2DM) was diagnosed according to the American Diabetes Association guidelines. The MetS was diagnosed according to the National Cholesterol Education Program ATP-III criteria (NCEP-ATP-III), and hypertension according to the NCEP-ATP-III criteria for high blood pressure therein or anti-hypertensive treatment. Body mass index (BMI) was calculated as body weight (kg)/height^2^ (m) and obesity was diagnosed at BMI ≥ 30. Venous blood samples were collected after an overnight fast of 12 h. Basic laboratory measurements were immediately performed from fresh serum samples. Aliquots of these samples were frozen and stored at −80°C and used for liquid chromatography-tandem mass spectrometry (LC-MS/MS). LDL-C and high-density lipoprotein cholesterol (HDL-C) were measured using enzymatic hydrolysis and precipitation techniques on a Hitachi-Analyzer 717 or 911 (QuantolipLDL, QuantolipHDL; Roche, Basel, Switzerland). Remnant cholesterol (remnant C) concentration was calculated by subtracting LDL-C and HDL-C from total cholesterol (total C). Lipoprotein(a) [Lp(a)] was measured by particle-enhanced immunoturbidimetry (Tina-quant, Roche, Basel, Switzerland) on a Hitachi 717 or 911. Apolipoprotein (apo) A-I and apoB-100 were determined on a Cobas Integra 800 (Roche). As the cholesterol content of Lp(a) contributes approximately 30% to LDL-C mass,^26^ we additionally calculated corrected LDL-C (LDL-C_corr_): LDL-C_corr_ (mg/dL)=LDL-C (mg/dL) − 0.3 × Lp(a) (mg/dL). As a further alternative and instead of direct LDL-C measuring, we calculated LDL-C according to the recent Sampson-NIH equitation.^27^ The determination by LC-MS/MS analysis,^28^ the pre-selection of ceramides and phosphatidylcholines,^29^ and the combination of specific ceramides and phosphatidylcholines for calculating the scores coronary event risk test (CERT) and CERT2^24^ have been described previously.

### Statistical analysis

Normal distribution was checked using the Kolmogorov–Smirnov test. Non-normally distributed variables were described using median and IQR. Differences were tested for statistical significance with Chi-squared tests for categorical and with Jonckheere–Terpstra tests for continuous variables. Correlation analyses were performed by calculating non-parametric Spearman rank correlation coefficients. For prognostic endpoints, adjusted hazard ratios (HRs) were derived from Cox proportional hazards models. The proportional hazard assumption was checked by examination of scaled Schoenfeld residuals. No imputation was applied and all data were analysed by complete-case analysis. To examine the potential utility of predictive biomarkers, composed models were compared by calculating Harrell’s C and Somers’ D for right-censored data. All statistical analyses were performed with SPSS 28.0 for Windows (IBM Corp., USA), and R statistical software v. 4.2.2 (http://www.r-project.org).

## Results

### Patient characteristics

Patient characteristics are summarized in *Table 1*. The study comprised 1195 participants with a particularly high cardiovascular risk. Their median age was 67 years, and the median LDL-C concentration was 119 mg/dL (3.1 mmol/L). Among these 1195 patients, 20% were older than 75 years, 30% had T2DM, and 51% were taking statins at baseline. Together, 70% had at least one of these three characteristics, reflecting patient bias. Additionally, 89% had hypertension, 44% had metabolic syndrome, 28% were obese, and 19% were current smokers, accounting for 94% with at least one of these four characteristics. The median follow-up was 11.9 years, with a maximum of 16.1 years and an IQR of 7.5–13.8 years. Follow-up data were available for 1185 patients (>99% follow-up rate) comprising 12 262 person-years of follow-up. In total, 553 patients (47%) succumbed to death and 207 (17%) to cardiovascular death. Additionally, 399 (34%) patients experienced the MACE.

**Table 1:** Baseline patient characteristics

	Total	Lower LDL-C (<150 mg/dL)	High LDL-C (≥150 mg/dL)	*P*-value
	*n* = 1195	*n* = 907	*n* = 288	
Age, years	67 (58–74)	67 (59–74)	66 (57–72)	0.012
Age >75, no. (%)	241 (20)	196 (22)	45 (16)	0.027
Male sex, no. (%)	791 (66)	615 (68)	176 (61)	0.036
BMI, kg/m^2^	27 (25–30)	27 (25–30)	27 (25–30)	0.961
Obesity, no. (%)	331 (28)	255 (28)	76 (27)	0.583
Waist circumference, cm	98 (91–107)	99 (91–107)	98 (92–105)	0.275
LDL-C, mg/dL (mmol/L)	119 (92–148)	107 (85–126)	173 (161–195)	<0.001
	(3.1 (2.4–3.8))	(2.8 (2.2–3.3))	(4.5 (4.2–5.0))	
T2DM, no. (%)	358 (30)	297 (33)	61 (21)	<0.001
MetS, no. (%)	527 (44)	396 (44)	131 (45)	0.587
Hypertension, no. (%)	1069 (89)	811 (89)	258 (90)	0.936
Current smoking, no. (%)	231 (19)	177 (20)	54 (19)	0.775
Statin treatment, no. (%)	613 (51)	526 (58)	87 (30)	<0.001

Dichotomous data are given as proportion, continuous data (all not normally distributed) as median and interquartile range (IQR). Differences between the lower and high LDL-C subgroups were tested with Chi-squared tests for categorical and Jonckheere–Terpstra test for continuous variables.

### Association of LDL-C and ceramide markers with cardiovascular mortality, major cardiovascular events, and overall mortality

The association between cardiovascular mortality and the set of analysed lipid markers is outlined in detail in *Table 2*. Specifically, LDL-C showed a negative association with cardiovascular mortality [HR per 10 mg/dL = 0.94 (0.91–0.98)]. Virtually identical results were found for LDL-C corrected for the LP(a) content [LDL_corr_; HR per 10 mg/dL = 0.94 (0.91–0.97)] or calculated according to the Sampson-NIH equitation [LDL_calc_; HR per 10 mg/dL = 0.94 (0.91–0.98)]. This was also true regarding the secondary endpoints MACE and overall mortality (see Supplementary material online, *Table S1*). Ceramides Cer(d18:1/16:0), Cer(d18:1/18:0), Cer(d18:1/24:1) and the phosphatidylcholine PC32:0 were positively associated with outcomes cardiovascular mortality, overall mortality, and MACE, whereas ceramide Cer(d18:1/24:0) and phosphatidylcholines PC36:6, PC:38:5 were negatively associated with these outcomes (*Table 2* and Supplementary material online, *Table S1*).

**Table 2:** Hazard ratios and c-statistics for lipid predictors of cardiovascular mortality

	Parameter	HR	95% CI	*P*	Harrel's C	Somers’ D	AUC	95%CI	*P*
Cholesterol and routine lipid markers	Cardiovascular mortality								
	LDL-C	0.78 (0.94)^a^	0.68–0.91	0.001	0.428	−0.145	0.559^b^	0.515–0.602^b^	0.008
	Lp(a)	1.11 (1.02)^a^	0.98–1.26	0.114	0.551	0.101	0.535^b^	0.491–0.579^b^	0.120
	LDL-C_corr_	0.76 (0.94)^a^	0.66–0.89	<0.001	0.413	−0.174	0.568^b^	0.523–0.613^b^	0.003
	LDL-C_calc_	0.80 (0.94)^a^	0.69–0.92	0.002	0.435	−0.130	0.554^b^	0.512–0.597^b^	0.013
	HDL-C	0.78 (0.86)^a^	0.67–0.91	0.001	0.430	−0.140	0.558^b^	0.514–0.603^b^	0.008
	Total C	0.77 (0.95)^a^	0.67–0.90	<0.001	0.423	−0.154	0.561^b^	0.517–0.604^b^	0.006
	Remnant C	1.15 (1.09)^a^	1.04–1.28	0.006	0.562	0.124	0.557	0.513–0.600	0.011
	Triglycerides	1.05 (1.01)^a^	0.93–1.20	0.415	0.504	0.071	0.516	0.472–0.560	0.472
	apoB-100	0.89 (0.95)^a^	0.77–1.02	0.097	0.457	−0.086	0.532^b^	0.489–0.575^b^	0.145
	apoA-1	0.78 (0.92)^a^	0.67–0.91	0.001	0.424	−0.153	0.556^b^	0.512–0.601^b^	0.011
Ceramides and phosphatidylcholines	Cer(d18:1/16:0)	1.17	1.03–1.32	0.016	0.551	0.102	0.537	0.493–0.580	0.098
	Cer(d18:1/18:0)	1.10	0.97–1.25	0.148	0.540	0.079	0.530	0.486–0.573	0.181
	Cer(d18:1/24:0)	0.76	0.66–0.89	<0.001	0.425	−0.149	0.562^b^	0.518–0.605^b^	0.005
	Cer(d18:1/24:1)	1.23	1.08–1.39	0.001	0.571	0.141	0.561	0.517–0.604	0.006
	PC 32:0	1.11	0.97–1.28	0.114	0.519	0.039	0.525	0.480–0.570	0.259
	PC 36:6	0.66	0.56–0.78	<0.001	0.371	−0.258	0.601^b^	0.558–0.645^b^	<0.001
	PC 38:5	0.91	0.79–1.06	0.212	0.446	−0.109	0.523^b^	0.479–0.568^b^	0.288
Ratios and scores of ceramides and phosphatidylcholines	Cer(d18:1/16:0)/Cer(d18:1/24:0)	1.17	1.12–1.23	<0.001	0.648	0.295	0.620	0.580–0.660	<0.001
	Cer(d18:1/18:0)/Cer(d18:1/24:0)	1.28	1.18–1.39	<0.001	0.603	0.207	0.579	0.537–0.621	<0.001
	Cer(d18:1/24:1)/Cer(d18:1/24:0)	1.17	1.12–1.23	<0.001	0.656	0.312	0.628	0.588–0.668	<0.001
	Cer(d18:1/18:0)/PC 36:6	1.36	1.25–1.48	<0.001	0.627	0.254	0.594	0.551–0.636	<0.001
	Cer(d18:1/24:1)/PC 36:6	1.51	1.39–1.64	<0.001	0.663	0.325	0.628	0.587–0.670	<0.001
	CERT	1.52 (1.14)^c^	1.33–1.73	<0.001	0.623	0.247	0.600	0.557–0.643	<0.001
	CERT2	1.74 (1.24)^c^	1.51–2.00	<0.001	0.662	0.323	0.623	0.581–0.665	<0.001

The result of Cox regression analysis and evaluation of proposed parameters are summarized with hazard ratios (HRs), Harrels's C, Somers’ D, and areas under the curve (AUC) of receiver operating characteristics (ROC).

HRs are given per one standard deviation (SD), and alternatively ^a^ per 10 mg/dL increase, or ^c^ per 1 higher score (CERT, CERT2).

^b^AUC < 0.5 was transformed by AUC-1 for easy comparison.

In general, HRs for the mentioned endpoints were higher when combinations of ceramides or combinations of ceramides and phosphatidylcholines were analysed. Based on Harrels’ C or Somers’ D, which assess the discrimination in survival analysis, the ratio Cer(d18:1/24:1)/PC36:6 and the score CERT2 exhibited the highest predictive power among all analysed markers (*Table 2* and Supplementary material online, *Table S1*).

### Risk curves differ between LDL-c and ceramide markers

**Figure 1:**
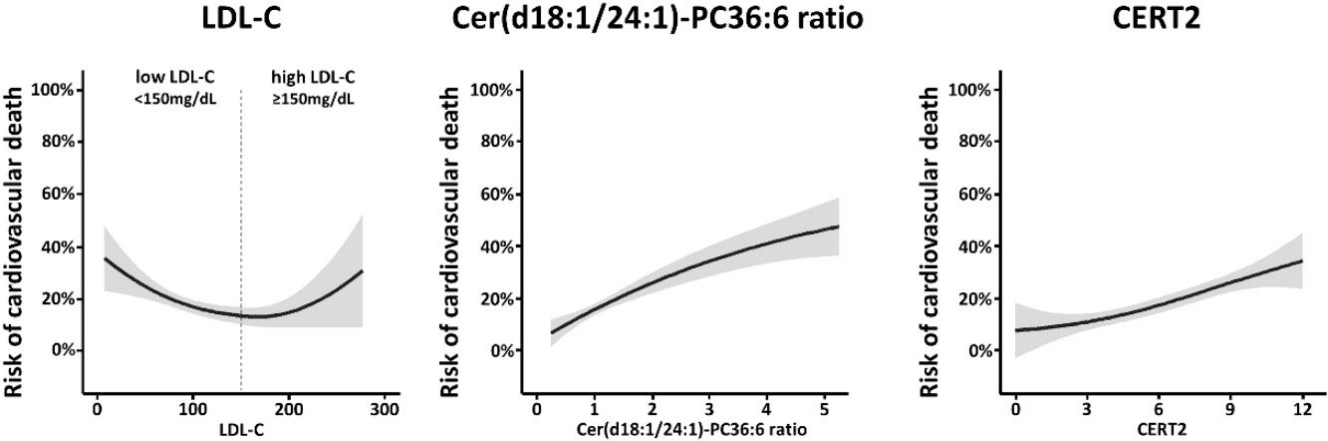
Risk curves for cardiovascular mortality of LDL-C, the ratio Cer(d18:1/24:1)/PC 36:6, and CERT2. The risk curves are calculated according to loess (LOcally WEighted Scatter-plot Smoother) with 95% confidence intervals (grey) for the risk of cardiovascular death. LDL-C is given as mg/dL, the subgroups of patients with either low (<150 mg/dL) or high LDL-C at baseline (≥150 mg/dL) are separated by a dashed line. The ratio Cer(d18:1/24:1)/PC 36:6 is given as µmol/(L × peak intensity), and CERT2 is given as a score ranging from 0 to 12. Data were obtained from single human samples.

A comprehensive overview of risk curves for all endpoints and lipid markers is depicted in Supplementary material online, *Figure S1*. Focusing on LDL-C and the highly predictive ceramide markers, we found that the risk curve for cardiovascular mortality demonstrated a U-shaped relation along with increasing LDL-C as illustrated in *Figure 1*. A similar curve was observed for MACE. For overall death, we found an inverse relation (see Supplementary material online, *Figure S1*). In contrast to LDL-C, we found no U-shaped or inverse relation between the fatal outcomes and Cer(d18:1/24:1)/PC 36:6 or CERT2 but rather a linear relation (*Figure 1* and Supplementary material online, *Figure S1*). Moreover, the relationship between LDL-C and these ceramide-based markers was not linear but displayed a reverse U-shaped pattern as depicted in scatter plots in Supplementary material online, *Figure S2*. This demonstrates that LDL-C does not increase with higher levels of these markers.

### Association of lipid markers with cardiovascular mortality in patients with either high or low LDL-C

Given the U-shape found for the relation between LDL-C and cardiovascular outcome in our study (*Figure 1*) and data from the literature mentioned above describing a vertex for LDL-C concentrations of around 150 mg/dL LDL-C,^9,20–22^ we stratified our study population into high and lower LDL-C subgroups based on a threshold of 150 mg/dL (≥150 mg/dL, *n* = 288 vs. < 150 mg/dL, *n* = 907). Comparing both subgroups, patients with lower LDL-C readings at baseline had a significantly higher mean age, a significantly higher prevalence of male sex and T2DM, and were significantly more often under statin treatment at baseline than the group with higher LDL-C readings (*Table 1*).

**Figure 2:**
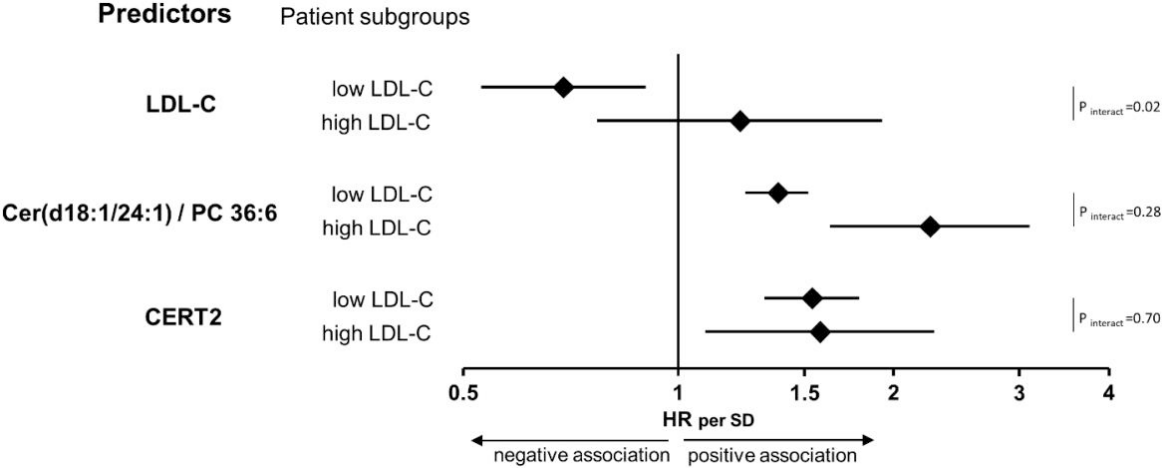
Association of cardiovascular mortality and LDL-C, the ceramide-based ratio Cer(d18:1/24:1)/PC 36:6, and the score CERT2 in patients with either high or low LDL-C. The Forest plots represent the adjusted hazard ratios (HR) of cardiovascular mortality by applying a Cox regression model in subgroups of patients with either lower (<150 mg/dL) or high LDL-C at baseline (≥150 mg/dL). The model includes the covariates age, sex, the status of type 2 diabetes mellitus (T2DM), and the status of statin treatment at baseline as covariates. The HR is given per 1 SD together with the 95% confidence interval. The *P*-value for interaction between the high/lower LDL-C group variable and LDL-C, the ratio Cer(d18:1/24:1)/PC 36:6 or CERT2 is indicated. Data were obtained from single human samples.

In a multivariate Cox regression model, adjusting for the above-mentioned variables age, sex, T2DM status, and statin treatment status, we observed a clear difference between the high and lower LDL-C subgroups with most lipid markers in terms of risk prediction (see Supplementary material online, *Table S2*). This was particularly evident for LDL-C. The risk for cardiovascular mortality increased by 24% per 1 SD higher LDL-C in the high LDL-C subgroup but decreased by 34% in the low LDL-C subgroup, though both results were not significant. Only combinations of ceramides and ceramides with phosphatidylcholines [Cer(d18:1/24:1)/Cer(d18:1/24:0), Cer(d18:1/18:0)/PC 36:6, Cer(d18:1/24:1)/PC 36:6, CERT and CERT2] as well as remnant cholesterol were able to significantly predict the cardiovascular mortality risk in both subgroups (see Supplementary material online, *Table S2*). *Figure 2* illustrates that, in sharp contrast to LDL-C, CERT2 almost identically predicted cardiovascular mortality in the high and lower LDL-C groups, demonstrating a significant 58% increase per 1 SD higher CERT2 in the high LDL-C group and a significant 54% increase in the group with lower LDL-C.

Finally, as the mentioned variables (age, sex, T2DM status, and statin treatment status) differed significantly between the lower and high LDL-C subgroups, we further stratified patients based on age (≤75 vs. > 75 years), sex, T2DM, and statin use at baseline and also based on the type of ASCVD manifestation (PAD patients vs. CAG patients) and other LDL-C thresholds (100 mg/dL, 70 mg/dL). Similar to the results after initial LDL-C stratification (150 mg/dL), we found that, applying all these types of stratification, only the ratio Cer(d18:1/24:1)/PC 36:6 and CERT2 but not LDL-C were significantly associated with the outcome in both subgroups (see Supplementary material online, *Figure S3*).

## Discussion

In our study, which comprised 1195 cardiovascular disease patients and encompassed 12 262 patient-years, we identified a U-shaped association between LDL-C and cardiovascular mortality risk. Notably, the association between LDL-C and cardiovascular mortality differed between patients with lower LDL-C (demonstrating a negative trend) and those with higher LDL-C (demonstrating a positive trend). In contrast, when analysing ceramide- and phosphatidylcholine-based markers, specifically the ratio Cer(d18:1/24:1)/PC 36:6 and the CERT2 score, we identified a linear increase in cardiovascular risk. These markers emerged as particularly powerful risk predictors in patients with either lower or high LDL-C levels.

Recent large studies in Korea by Sung *et al.* and in Denmark by Johannesen *et al.* consistently found that both high and low levels of LDL-C were associated with an increased risk of all-cause and cardiovascular mortality, with the lowest risk observed around an LDL-C concentration of 140–150 mg/dL.^20,21^ Our study also yielded similar results. In addition to LDL-C, a U-shaped risk curve has been identified in numerous studies for the association between mortality and total cholesterol^6,30–32^ or HDL-C.^33,34^ In the case of HDL-C, some study authors have suggested that using HDL-C as a tool for cardiovascular risk prediction may no longer be an effective clinical strategy.^34^ We believe that this is also true for LDL-C.

Many studies have shown that ageing exerts a notable impact on LDL-C levels.^35–39^ Therefore, advanced age, which is often accompanied by comorbidities and medical treatment, poses a significant constraint on risk prediction in the context of our ageing society.^7^ In a recent study, we demonstrated that cholesterol readings in patients with a mean age of 65 years were significantly less valuable compared to readings obtained earlier in their lives when the same patients were younger, healthier, and not undergoing medical treatment.^8^ These findings align with previous Copenhagen city study data, which have suggested that genotype, which is independent of age, is a better predictor of risk than LDL-C concentration in adult life.^40^ Taken together, these data provide further support for previous studies that have reported an absent or even inverse causal relation between total cholesterol or LDL-C and cardiovascular disease in old age.^30,41,42^

In addition to ageing, diseases and medical treatment are two further well-known factors that impact cholesterol levels.^5–8^ Data from the Copenhagen General Population Study have revealed that comorbidities were more frequent in individuals with the lowest levels of LDL-C.^20^ In our study, the low LDL-C group not only had an older age profile but also had a higher percentage of diabetic patients and statin users. Previous data from the MIRACL trial have yet demonstrated that LDL-C was not predictive for future cardiovascular events in patients hospitalized for acute coronary syndrome.^15^ Similarly, the PESA study revealed that atherosclerosis is often associated with LDL-C concentration within the normal range.^16^ Furthermore, a comprehensive analysis of over 135 000 hospitalizations with coronary artery disease in the USA as part of the Get With The Guidelines programme indicated that these patients had lower LDL-C compared to the general population.^14^ The JUPITER trial, based on the fact that half of all myocardial infarctions and strokes occur among patients with levels of LDL-C below treatment thresholds,^43^ ultimately showed that rosuvastatin reduced the incidence of MACE in those patients with LDL-C below the treatment threshold.^43^ Recent research by Ridker *et al.* further supports the above-mentioned findings, as they found no association between LDL-C and MACE in statin-treated patients at high ASCVD risk.^10^ However, they did identify a clear association between high-sensitive CRP and cardiovascular risk,^10^ suggesting that factors other than cholesterol may be better predictors. Similarly, Johannesen *et al.* suggested that LDL-C should be interpreted in combination with other risk factors rather than solely relying on its measured value when making clinical decisions.^20^ They found that the lowest risk was observed at an LDL-C level of 140 mg/dL in their study and argued that in individuals with an otherwise low risk of ASCVD, such a moderate increase in LDL-C levels may not be hazardous on its own, and treatment should not be initiated based solely on this criterion.^20^ While they have emphasized that this pertains to the general population and is not specifically centred on cardiovascular disease, it diverges from ‘the lower the better’ principle.^44^ Considering the expanding body of evidence, it is evident that a more comprehensive cardiovascular risk assessment, beyond the use of LDL-C, is necessary for accurate risk prediction and treatment decisions, especially in populations with advanced age, comorbidities, and existing statin treatment.

The present study demonstrates that ceramide- and phosphatidyl-choline-based markers and scores provide greater predictive capability and consistency across subgroups defined by age, sex, diabetes, LDL-C levels, and statin treatment compared to LDL-C. Ceramides and phosphatidylcholines exhibit distinct distribution patterns in lipoprotein particles,^45^ and previous research has demonstrated that their combination synergistically enhances risk prediction.^24^ In both primary and secondary prevention, ceramide and phosphatidylcholine scores have proven effective in predicting cardiovascular risk.^46,47^ Nevertheless, recent suggestions highlight the need for further studies before considering the integration of ceramide- and phosphatidylcholine-based scores into routine clinical practice.^48^ Our present study emphasizes the advantages of using different lipid class markers and identifies the combination of ceramides and phosphatidylcholines as the most potent predictors. In the context explored here, ceramide-based markers have also shown clear superiority over LDL-C, aligning with previous findings from the WECAC, LIPID, and Karola trials.^24^ Despite these promising findings, the full extent to which ceramide levels are impacted by various imponderables remains uncertain. Unlike LDL-C, statins do not directly block ceramide synthesis. However, there is a certain indirect statin effect on ceramide levels,^49^ and ceramide levels, on the other hand, might probably influence the protective effects of statin treatment.^50^ Therefore, the ongoing development of drugs targeting ceramide levels suggests that the value of ceramides in risk prediction may be reconsidered in the future.

Our study’s data shed light on the limitations of LDL-C as a predictor of cardiovascular risk in real-world populations. LDL-C failed to predict cardiovascular mortality at once in low and high LDL-C subgroups. Notably, we observed no improvement in risk prediction when subtracting Lp(a) from LDL-C, or when using calculated instead of measured LDL-C levels. Additionally, apart from remnant cholesterol, none of the other lipid species namely Lp(a), HDL-C, total C, triglycerides, ApoA-1, and ApoB-100 were successful in predicting the outcome after LDL-C stratification. That said, it is essential to clarify that a causal risk factor is different from a risk predictor and the lack of predictive power for LDL-C in this study should not be misconstrued as questioning the well-established causal relationship between LDL-C and ASCVD. LDL-C is the most important causal cardiovascular risk factor, and we firmly support the use of statins to efficiently reduce cardiovascular risk by lowering LDL-C levels.^1–4^

### Strengths and limitations

This study has strengths and limitations. A particular strength of this study is the 99% follow-up rate. An additional strength is, that the study comprises a very well-characterized patient cohort and that all patients were in a stable stage. Furthermore, we performed a prospective study comprising 12 262 patient-years in a cohort with a high cardiovascular risk, which deserves particular clinical attention.

One limitation is the fact that we selected Caucasian patients with cardiovascular disease only. Consequently, the mean annual cardiovascular death rate was 1.7%, which is about four times higher than in the general population of the EU (0.4%).^51^ Therefore, the results are not representative in view of the general population nor necessarily applicable to other patients or other ethnicities. Furthermore, our analyses are based on single measurements. We did not include measurements at different time points and thus could not preclude that changes in lipid levels might have impacted the outcome. Finally, we have data on the prescription of drugs but not on adherence to the respective medical treatment, which also may impact the outcome.

## Conclusion

Advanced age, comorbidities, and medical treatment are known confounders of LDL-C concentration in patients. The present study poses the issue of LDL-C controversies and it also brings up the importance of additional biomarkers of ASCVD risk—namely ceramides and phosphatidylcholines that may weigh heavily when assessing the risk profile of a particular patient. It demonstrates that ceramide- and phosphatidylcholine-containing markers provide more accurate cardiovascular risk prediction than LDL-C. Regarding clinical importance, this study has shown that these markers, and in particular CERT2, are valuable risk predictors, even in a population in which LDL-C fails. Other than LDL-C or further cholesterol-based markers like HDL-C or total cholesterol, a low value of CERT2 score reflects a low risk, and a high value reflects a high risk. This association is independent of LDL-C levels. Even in a population in which LDL-C and outcome are U-shaped or inversely associated, the risk for cardiovascular mortality increases in a linear way with increasing CERT2. This enables a more precise risk prediction for high-risk patients.

## Supplementary Material

oeae001_Supplementary_Data

## Data Availability

The data that support the findings of this study are available from the corresponding author upon reasonable request.

## Lead author biography

Andreas Leiherer holds a Doctorate in Natural Sciences (Dr rer. nat.) from the University of Regensburg (Germany) and a Doctorate in Medical Science (Dr scient. med.) from the Private University in the Principality of Liechtenstein. He earned his venia docendi (Priv.-Doz.) in Experimental Internal Medicine from the Medical University Innsbruck (Austria). Currently, he serves as the Vice-Chairperson of the Vorarlberg Institute of Vascular Investigation and Treatment (VIVIT) Laboratories, an Austrian non-profit research institute. Leiherer has extensive expertise in data analysis, statistics, and epidemiology, with a current focus on using large datasets for advanced risk prediction. His research encompasses various fields, including cardiovascular and metabolic diseases, virology, and genetics.
