# Supplementary material for: Ceramides improve cardiovascular risk prediction beyond low-density lipoprotein cholesterol
Source: Eur Heart J Open. 2024 Jan 8;4(1):oeae001. doi: 10.1093/ehjopen/oeae001 (PMC10826640; doi:10.1093/ehjopen/oeae001)
Supplement: oeae001_Supplementary_Data [file oeae001_supplementary_data.docx]

***Supplemental data***

***Supplementary table 1. Hazard ratios and C-statistics of lipid predictors of MACE and overall mortality.***

|  | **Parameter** | **HR** | **95%CI** | **p** | **Harrel’s C** | **Somers‘ D** | **AUC** | **95%CI** | **p** |
| --- | --- | --- | --- | --- | --- | --- | --- | --- | --- |
|  |  | | | | | | | | |
|  | **MACE** | | | | | | | | |
| Cholesterol and routine lipid markers | LDL-C | 0.86 (0.97)^#^ | 0.78-0.96 | 0.006 | 0.451 | -0.097 | 0.536* | 0.501-0.571* | 0.043 |
|  | Lp(a) | 1.05 (1.01) ^#^ | 0.95-1.15 | 0.373 | 0.529 | 0.058 | 0.527 | 0.492-0.563 | 0.131 |
|  | LDL-C_corr_ | 0.85 (0.96) ^#^ | 0.76-0.94 | 0.003 | 0.445 | -0.110 | 0.540* | 0.505-0.576* | 0.026 |
|  | LDL-C_calc_ | 0.87 (0.96) ^#^ | 0.79-0.97 | 0.009 | 0.457 | -0.086 | 0.533* | 0.499-0.568* | 0.057 |
|  | HDL-C | 0.77 (0.86) ^#^ | 0.69-0.86 | <0.001 | 0.423 | -0.154 | 0.573* | 0.539-0.608* | <0.001 |
|  | Total C | 0.85 (0.97)^#^ | 0.76-0.94 | 0.002 | 0.447 | -0.106 | 0.543 | 0.509-0.578* | 0.014 |
|  | remnant C | 1.16 (1.09) ^#^ | 1.08-1.25 | <0.001 | 0.557 | 0.114 | 0.553 | 0.518-0.588 | 0.0035 |
|  | triglycerides | 1.11 (1.01) ^#^ | 1.01-1.21 | 0.023 | 0.531 | 0.063 | 0.541 | 0.506-0.576 | 0.021 |
|  | apoB-100 | 0.96 (0.98) ^#^ | 0.87-1.06 | 0.436 | 0.482 | -0.036 | 0.505* | 0.470-0.540* | 0.775 |
|  | apoA-1 | 0.77 (0.91) ^#^ | 0.67-0.86 | <0.001 | 0.423 | -0.154 | 0.567* | 0.532-0.601* | <0.001 |
|  | | | | | | | | | |
| Ceramides and PCs | Cer(d18:1/16:0) | 1.14 | 1.03-1.25 | 0.009 | 0.530 | 0.061 | 0.529 | 0.494-0.565 | 0.100 |
|  | Cer(d18:1/18:0) | 1.10 | 1.01-1.21 | 0.039 | 0.523 | 0.045 | 0.528 | 0.493-0.563 | 0.118 |
|  | Cer(d18:1/24:0) | 0.93 | 0.84-1.03 | 0.175 | 0.467 | -0.065 | 0.505* | 0.470-0.540* | 0.783 |
|  | Cer(d18:1/24:1) | 1.19 | 1.08-1.30 | <0.001 | 0.553 | 0.107 | 0.556 | 0.521-0.591 | 0.002 |
|  | PC 32:0 | 1.05 | 0.94-1.16 | 0.392 | 0.506 | 0.011 | 0.508 | 0.473-0.543 | 0.643 |
|  | PC 36:6 | 0.84 | 0.76-0.94 | 0.002 | 0.430 | -0.140 | 0.541* | 0.506-0.576* | 0.022 |
|  | PC 38:5 | 0.94 | 0.84-1.04 | 0.239 | 0.462 | -0.076 | 0.508* | 0.473-0.543* | 0.645 |
|  | | | | | | | | | |
| Ratios and scores of ceramides and PCs | Cer(d18:1/16:0) / Cer(d18:1/24:0) | 1.10 | 1.05-1.16 | <0.001 | 0.579 | 0.159 | 0.545 | 0.511-0.580 | 0.011 |
|  | Cer(d18:1/18:0) / Cer(d18:1/24:0) | 1.15 | 1.06-1.24 | <0.001 | 0.551 | 0.102 | 0.531 | 0.496-0.565 | 0.086 |
|  | Cer(d18:1/24:1) / Cer(d18:1/24:0) | 1.11 | 1.06-1.16 | <0.001 | 0.591 | 0.182 | 0.559 | 0.525-0.593 | 0.001 |
|  | Cer(d18:1/18:0) / PC 36:6 | 1.25 | 1.16-1.34 | <0.001 | 0.570 | 0.140 | 0.545 | 0.510-0.580 | 0.012 |
|  | Cer(d18:1/24:1) / PC 36:6 | 1.34 | 1.25-1.45 | <0.001 | 0.594 | 0.188 | 0.564 | 0.530-0.599 | <0.001 |
|  |  |  |  |  |  |  |  |  |  |
|  | CERT | 1.28 (1.08)^3^ | 1.17-1.41 | <0.001 | 0.572 | 0.144 | 0.556 | 0.521-0.590 | 0.002 |
|  | CERT2 | 1.34 (1.12)^3^ | 1.21-1.48 | <0.001 | 0.585 | 0.170 | 0.547 | 0.512-0.582 | 0.008 |
|  | | | | | | | | | |
| **Overall mortality** | | | | | | | | | |
| Cholesterol and routine lipid markers | LDL-C | 0.80 (0.95) ^#^ | 0.73-0.87 | <0.001 | 0.439 | -0.122 | 0.573* | 0.541-0.606* | <0.001 |
|  | Lp(a) | 0.99 (1.00) ^#^ | 0.91-1.08 | 0.872 | 0.512 | 0.024 | 0.513* | 0.479-0.547* | 0.449 |
|  | LDL-C_corr_ | 0.80 (0.95) ^#^ | 0.73-0.87 | <0.001 | 0.431 | -0.138 | 0.573* | 0.539-0.606* | <0.001 |
|  | LDL-C_calc_ | 0.82 (0.95) ^#^ | 0.75-0.89 | <0.001 | 0.446 | -0.108 | 0.568* | 0.536-0.601* | <0.001 |
|  | HDL-C | 0.89 (0.93) ^#^ | 0.82-0.98 | 0.013 | 0.462 | -0.076 | 0.527* | 0.494-0.560* | 0.103 |
|  | Total C | 0.80 (0.95)^#^ | 0.73-0.87 | <0.001 | 0.438 | -0.123 | 0.574* | 0.541-0.606* | <0.001 |
|  | remnant C | 1.07 (1.04) ^#^ | 1.00-1.16 | 0.061 | 0.528 | 0.057 | 0.514 | 0.480-0.547 | 0.423 |
|  | triglycerides | 0.97 (1.00) ^#^ | 0.89-1.06 | 0.523 | 0.481 | -0.038 | 0.531* | 0.498-0.564* | 0.069 |
|  | apoB-100 | 0.86 (0.94) ^#^ | 0.79-0.94 | <0.001 | 0.457 | -0.085 | 0.554* | 0.521-0.587* | 0.001 |
|  | apoA-1 | 0.85 (0.95) ^#^ | 0.78-0.93 | <0.001 | 0.443 | -0.114 | 0.548* | 0.515-0.581* | 0.004 |
|  | | | | | | | | | |
| Ceramides and PCs | Cer(d18:1/16:0) | 1.17 | 1.08-1.26 | <0.010 | 0.549 | 0.098 | 0.541 | 0.509-0.574 | 0.014 |
|  | Cer(d18:1/18:0) | 1.14 | 1.06-1.23 | <0.001 | 0.537 | 0.074 | 0.540 | 0.507-0.573 | 0.018 |
|  | Cer(d18:1/24:0) | 0.78 | 0.71-0.86 | <0.001 | 0.431 | -0.137 | 0.587* | 0.555-0.619* | <0.001 |
|  | Cer(d18:1/24:1) | 1.21 | 1.12-1.31 | <0.001 | 0.562 | 0.123 | 0.566 | 0.533-0.598 | <0.001 |
|  | PC 32:0 | 1.10 | 1.01-1.20 | 0.023 | 0.526 | 0.052 | 0.531 | 0.498-0.564 | 0.068 |
|  | PC 36:6 | 0.68 | 0.61-0.75 | <0.001 | 0.392 | -0.215 | 0.625* | 0.593-0.656* | <0.001 |
|  | PC 38:5 | 0.84 | 0.77-0.93 | <0.001 | 0.440 | -0.120 | 0.568* | 0.535-0.601* | <0.001 |
|  | | | | | | | | | |
| Ratios and scores of ceramides and PCs | Cer(d18:1/16:0) / Cer(d18:1/24:0) | 1.17 | 1.13-1.21 | <0.001 | 0.638 | 0.276 | 0.659 | 0.628-0.690 | <0.001 |
|  | Cer(d18:1/18:0) / Cer(d18:1/24:0) | 1.27 | 1.21-1.34 | <0.001 | 0.603 | 0.206 | 0.626 | 0.594-0.658 | <0.001 |
|  | Cer(d18:1/24:1) / Cer(d18:1/24:0) | 1.17 | 1.13-1.20 | <0.001 | 0.646 | 0.292 | 0.671 | 0.640-0.701 | <0.001 |
|  | Cer(d18:1/18:0) / PC 36:6 | 1.35 | 1.27-1.42 | <0.001 | 0.617 | 0.234 | 0.637 | 0.606-0.668 | <0.001 |
|  | Cer(d18:1/24:1) / PC 36:6 | 1.47 | 1.39-1.55 | <0.001 | 0.643 | 0.286 | 0.665 | 0.634-0.696 | <0.001 |
|  |  |  |  |  |  |  |  |  |  |
|  | CERT | 1.47 (1.13) ^$^ | 1.35-1.59 | <0.001 | 0.616 | 0.232 | 0.627 | 0.595-0.659 | <0.001 |
|  | CERT2 | 1.74 (1.24) ^$^ | 1.59-1.89 | <0.001 | 0.656 | 0.312 | 0.676 | 0.645-0.706 | <0.001 |

*The result of Cox regression analysis and evaluation of proposed parameters are summarizes with Hazard ratios (HR), Harrels’s C, Somers’D, and areas under the curve (AUC) of receiver operating characteristics (ROC). HRs are given per one standard deviation (SD). AUC <0.5 was transformed by AUC-1 for easy comparison.*

***Supplementary Table 2. Hazard ratios of lipid predictors in patients with low LDL-C or high LDL-C.***

|  | **Parameter** |  | **Low LDL-C** | | |  | **High LDL-C** | | |
| --- | --- | --- | --- | --- | --- | --- | --- | --- | --- |
|  |  | **Model** | **HR** | **95%CI** | **p** |  | **HR** | **95%CI** | **p** |
|  |  | | | | | | | | |
|  | **Cardiovascular mortality** | | | | | | | | |
| Cholesterol and routine lipid markers | LDL-C | univariate | 0.69 | 0.55-0.88 | 0.003 |  | 1.06 | 0.65-1.71 | 0.828 |
|  |  | multivariate | 0.69 | 0.53-0.90 | 0.007 |  | 1.22 | 0.77-1.93 | 0.388 |
|  | Lp(a) | univariate | 1.13 | 0.98-1.30 | 0.082 |  | 1.02 | 0.75-1.39 | 0.894 |
|  |  | multivariate | 1.17 | 1.01-1.36 | 0.037 |  | 0.92 | 0.69-1.22 | 0.557 |
|  | LDL-C_corr_ | univariate | 0.67 | 0.53-0.84 | <0.001 |  | 1.02 | 0.65-1.60 | 0.933 |
|  |  | multivariate | 0.66 | 0.51-0.85 | 0.001 |  | 1.24 | 0.82-1.87 | 0.312 |
|  | LDL-C_calc_ | univariate | 0.80 | 0.63-1.00 | 0.051 |  | 0.72 | 0.42-1.23 | 0.232 |
|  |  | multivariate | 0.83 | 0.65-1.06 | 0.136 |  | 0.88 | 0.52-1.49 | 0.636 |
|  | HDL-C | univariate | 0.76 | 0.64-0.90 | 0.001 |  | 0.95 | 0.67-1.36 | 0.794 |
|  |  | multivariate | 0.78 | 0.65-0.94 | 0.008 |  | 0.91 | 0.53-1.24 | 0.333 |
|  | Total C | univariate | 0.69 | 0.55-0.85 | <0.001 |  | 1.20 | 0.75-1.93 | 0.439 |
|  |  | multivariate | 0.73 | 0.58-0.93 | 0.009 |  | 1.37 | 0.86-2.17 | 0.186 |
|  | remnant C | univariate | 1.09 | 0.97-1.24 | 0.150 |  | 1.58 | 1.21-2.06 | <0.001 |
|  |  | multivariate | 1.15 | 1.00-1.32 | 0.043 |  | 1.77 | 1.36-2.32 | <0.001 |
|  | triglycerides | univariate | 0.96 | 0.82-1.13 | 0.643 |  | 1.57 | 1.22-2.02 | <0.001 |
|  |  | multivariate | 0.99 | 0.84-1.16 | 0.865 |  | 2.06 | 1.49-2.84 | <0.001 |
|  | apoB-100 | univariate | 0.91 | 0.73-1.13 | 0.379 |  | 1.17 | 0.81-1.68 | 0.410 |
|  |  | multivariate | 0.91 | 0.71-1.38 | 0.384 |  | 1.28 | 0.89-1.85 | 0.182 |
|  | apoA-1 | univariate | 0.74 | 0.63-0.88 | <0.001 |  | 1.10 | 0.78-1.54 | 0.596 |
|  |  | multivariate | 0.77 | 0.64-0.92 | 0.004 |  | 0.93 | 0.64-1.34 | 0.697 |
|  | | | | | | | | | |
| Ceramides and PCs | Cer(d18:1/16:0) | univariate | 1.16 | 1.01-1.34 | 0.033 |  | 1.57 | 1.14-2.16 | 0.006 |
|  |  | multivariate | 1.17 | 1.01-1.35 | 0.038 |  | 1.82 | 1.32-2.51 | <0.001 |
|  | Cer(d18:1/18:0) | univariate | 1.07 | 0.93-1.24 | 0.327 |  | 1.34 | 1.00-1.80 | 0.050 |
|  |  | multivariate | 1.13 | 0.96-1.33 | 0.133 |  | 1.46 | 1.07-2.00 | 0.019 |
|  | Cer(d18:1/24:0) | univariate | 0.69 | 0.56-0.84 | <0.001 |  | 1.08 | 0.82-1.43 | 0.593 |
|  |  | multivariate | 0.73 | 0.60-0.89 | 0.002 |  | 1.33 | 0.98-1.80 | 0.070 |
|  | Cer(d18:1/24:1) | univariate | 1.16 | 1.00-1.35 | 0.052 |  | 1.73 | 1.34-2.23 | <0.001 |
|  |  | multivariate | 1.10 | 0.94-1.30 | 0.247 |  | 1.57 | 1.21-2.05 | <0.001 |
|  | PC 32:0 | univariate | 1.11 | 0.94-1.31 | 0.216 |  | 1.50 | 1.12-2.01 | 0.007 |
|  |  | multivariate | 1.11 | 0.94-1.32 | 0.216 |  | 1.35 | 1.00-1.82 | 0.047 |
|  | PC 36:6 | univariate | 0.61 | 0.50-0.74 | <0.001 |  | 0.92 | 0.67-1.27 | 0.627 |
|  |  | multivariate | 0.67 | 0.55-0.81 | <0.001 |  | 1.02 | 0.72-1.43 | 0.093 |
|  | PC 38:5 | univariate | 0.79 | 0.66-0.95 | 0.012 |  | 1.44 | 1.10-1.87 | 0.008 |
|  |  | multivariate | 0.83 | 0.69-1.01 | 0.056 |  | 1.31 | 1.00-1.71 | 0.050 |
|  | | | | | | | | | |
| Ratios and scores of ceramides and PCs | Cer(d18:1/16:0) / Cer(d18:1/24:0) | univariate | 1.16 | 1.11-1.22 | <0.001 |  | 1.65 | 0.86-3.18 | 0.135 |
|  |  | multivariate | 1.13 | 1.07-1.19 | <0.001 |  | 1.48 | 0.72-3.05 | 0.290 |
|  | Cer(d18:1/18:0) / Cer(d18:1/24:0) | univariate | 1.26 | 1.15-1.37 | <0.001 |  | 1.45 | 0.92-2.28 | 0.105 |
|  |  | multivariate | 1.23 | 1.13-1.33 | <0.001 |  | 1.42 | 0.86-2.36 | 0.173 |
|  | Cer(d18:1/24:1) / Cer(d18:1/24:0) | univariate | 1.16 | 1.10-1.22 | <0.001 |  | 3.19 | 1.86-5.49 | <0.001 |
|  |  | multivariate | 1.12 | 1.06-1.18 | <0.001 |  | 1.83 | 1.08-3.09 | 0.025 |
|  | Cer(d18:1/18:0) / PC 36:6 | univariate | 1.32 | 1.20-1.44 | <0.001 |  | 1.94 | 1.42-2.65 | <0.001 |
|  |  | multivariate | 1.28 | 1.17-1.40 | <0.001 |  | 2.00 | 1.44-2.78 | <0.001 |
|  | Cer(d18:1/24:1) / PC 36:6 | univariate | 1.46 | 1.34-1.60 | <0.001 |  | 2.14 | 1.61-2.84 | <0.001 |
|  |  | multivariate | 1.38 | 1.24-1.52 | <0.001 |  | 2.25 | 1.63-3.10 | <0.001 |
|  |  |  |  |  |  |  |  |  |  |
|  | CERT | univariate | 1.48 | 1.28-1.71 | <0.001 |  | 1.73 | 1.24-2.40 | 0.001 |
|  |  | multivariate | 1.46 | 1.26-1.70 | <0.001 |  | 1.66 | 1.19-2.31 | 0.003 |
|  | CERT2 | univariate | 1.73 | 1.49-2.01 | <0.001 |  | 1.70 | 1.18-2.46 | 0.005 |
|  |  | multivariate | 1.54 | 1.32-1.79 | <0.001 |  | 1.58 | 1.09-2.28 | 0.016 |
|  | | | | | | | | | |
| **MACE** | | | | | | | | | |
| Cholesterol and routine lipid markers | LDL-C | univariate | 0.82 | 0.68-0.98 | 0.026 |  | 1.04 | 0.73-1.47 | 0.835 |
|  |  | multivariate | 0.86 | 0.71-1.05 | 0.135 |  | 1.23 | 0.88-1.71 | 0.221 |
|  | Lp(a) | univariate | 1.05 | 0.94-1.17 | 0.431 |  | 1.06 | 0.86-1.31 | 0.564 |
|  |  | multivariate | 1.06 | 0.95-1.19 | 0.297 |  | 0.98 | 0.80-1.19 | 0.817 |
|  | LDL-C_corr_ | univariate | 0.82 | 0.70-0.97 | 0.023 |  | 1.03 | 0.75-1.42 | 0.849 |
|  |  | multivariate | 0.85 | 0.71-1.02 | 0.090 |  | 1.24 | 0.92-1.67 | 0.165 |
|  | LDL-C_calc_ | univariate | 0.88 | 0.74-1.04 | 0.140 |  | 0.87 | 0.61-1.25 | 0.457 |
|  |  | multivariate | 0.94 | 0.78-1.13 | 0.497 |  | 1.09 | 0.77-1.55 | 0.616 |
|  | HDL-C | univariate | 0.78 | 0.69-0.88 | <0.001 |  | 0.75 | 0.58-0.97 | 0.030 |
|  |  | multivariate | 0.79 | 0.69-0.90 | <0.001 |  | 0.68 | 0.50-0.93 | 0.014 |
|  | Total C | univariate | 0.80 | 0.68-0.94 | 0.007 |  | 1.05 | 0.75-1.47 | 0.782 |
|  |  | multivariate | 0.87 | 0.73-1.03 | 0.111 |  | 1.25 | 0.89-1.75 | 0.207 |
|  | remnant C | univariate | 1.12 | 1.02-1.22 | 0.013 |  | 1.47 | 1.21-1.78 | <0.001 |
|  |  | multivariate | 1.16 | 1.06-1.28 | 0.002 |  | 1.53 | 1.24-1.88 | <0.001 |
|  | triglycerides | univariate | 1.05 | 0.95-1.16 | 0.328 |  | 1.42 | 1.18-1.71 | <0.001 |
|  |  | multivariate | 1.08 | 0.97-1.20 | 0.149 |  | 1.50 | 1.20-1.87 | <0.001 |
|  | apoB-100 | univariate | 1.03 | 0.88-1.21 | 0.698 |  | 1.14 | 0.88-1.47 | 0.334 |
|  |  | multivariate | 1.07 | 0.91-1.27 | 0.419 |  | 1.20 | 0.92-1.57 | 0.184 |
|  | apoA-1 | univariate | 0.78 | 0.69-0.88 | <0.001 |  | 0.76 | 0.59-0.99 | 0.044 |
|  |  | multivariate | 0.79 | 0.70-0.90 | <0.001 |  | 0.67 | 0.50-0.89 | 0.007 |
|  | | | | | | | | | |
| Ceramides and PCs | Cer(d18:1/16:0) | univariate | 1.15 | 1.04-1.28 | 0.005 |  | 1.31 | 1.04-1.66 | 0.023 |
|  |  | multivariate | 1.15 | 1.04-1.28 | 0.008 |  | 1.44 | 1.13-1.82 | 0.003 |
|  | Cer(d18:1/18:0) | univariate | 1.10 | 0.99-1.21 | 0.083 |  | 1.23 | 0.99-1.53 | 0.063 |
|  |  | multivariate | 1.12 | 1.00-1.26 | 0.045 |  | 1.21 | 0.96-1.52 | 0.105 |
|  | Cer(d18:1/24:0) | univariate | 0.93 | 0.81-1.06 | 0.262 |  | 1.07 | 0.87-1.30 | 0.532 |
|  |  | multivariate | 0.97 | 0.84-1.11 | 0.638 |  | 1.21 | 0.97-1.50 | 0.091 |
|  | Cer(d18:1/24:1) | univariate | 1.16 | 1.04-1.29 | 0.007 |  | 1.43 | 1.18-1.73 | <0.001 |
|  |  | multivariate | 1.13 | 1.01-1.27 | 0.035 |  | 1.30 | 1.07-1.57 | 0.007 |
|  | PC 32:0 | univariate | 1.04 | 0.92-1.18 | 0.502 |  | 1.26 | 1.01-1.56 | 0.037 |
|  |  | multivariate | 1.05 | 0.93-1.19 | 0.462 |  | 1.20 | 0.95-1.52 | 0.128 |
|  | PC 36:6 | univariate | 0.85 | 0.75-0.96 | 0.011 |  | 0.88 | 0.70-1.11 | 0.295 |
|  |  | multivariate | 0.91 | 0.80-1.03 | 0.123 |  | 0.93 | 0.73-1.19 | 0.557 |
|  | PC 38:5 | univariate | 0.87 | 0.76-0.98 | 0.026 |  | 1.24 | 1.02-1.51 | 0.029 |
|  |  | multivariate | 0.88 | 0.77-1.10 | 0.070 |  | 1.19 | 0.97-1.46 | 0.091 |
|  | | | | | | | | | |
| Ratios and scores of ceramides and PCs | Cer(d18:1/16:0) / Cer(d18:1/24:0) | univariate | 1.10 | 1.04-1.15 | <0.001 |  | 1.32 | 0.83-2.08 | 0.243 |
|  |  | multivariate | 1.07 | 1.01-1.13 | 0.015 |  | 1.18 | 0.73-1.90 | 0.504 |
|  | Cer(d18:1/18:0) / Cer(d18:1/24:0) | univariate | 1.23 | 1.04-1.23 | 0.004 |  | 1.27 | 0.92-1.77 | 0.150 |
|  |  | multivariate | 1.11 | 1.02-1.20 | 0.012 |  | 1.11 | 0.78-1.57 | 0.567 |
|  | Cer(d18:1/24:1) / Cer(d18:1/24:0) | univariate | 1.09 | 1.04-1.15 | <0.001 |  | 2.11 | 1.41-3.15 | <0.001 |
|  |  | multivariate | 1.07 | 1.01-1.13 | 0.027 |  | 1.40 | 0.94-1.07 | 0.097 |
|  | Cer(d18:1/18:0) / PC 36:6 | univariate | 1.21 | 1.11-1.31 | <0.001 |  | 1.72 | 1.33-2.22 | <0.001 |
|  |  | multivariate | 1.19 | 1.09-1.29 | <0.001 |  | 1.60 | 1.24-2.06 | <0.001 |
|  | Cer(d18:1/24:1) / PC 36:6 | univariate | 1.30 | 1.19-1.41 | <0.001 |  | 1.89 | 1.48-2.40 | <0.001 |
|  |  | multivariate | 1.24 | 1.14-1.36 | <0.001 |  | 1.82 | 1.41-2.35 | <0.001 |
|  |  |  |  |  |  |  |  |  |  |
|  | CERT | univariate | 1.27 | 1.15-1.42 | <0.001 |  | 1.36 | 1.08-1.71 | 0.009 |
|  |  | multivariate | 1.25 | 1.22-1.39 | <0.001 |  | 1.24 | 0.99-1.56 | 0.059 |
|  | CERT2 | univariate | 1.33 | 1.19-1.49 | <0.001 |  | 1.40 | 1.08-1.81 | 0.010 |
|  |  | multivariate | 1.24 | 1.11-1.39 | <0.001 |  | 1.23 | 0.95-1.59 | 0.110 |
|  | | | | | | | | | |
| **Overall mortality** | | | | | | | | | |
| Cholesterol and routine lipid markers | LDL-C | univariate | 0.76 | 0.66-0.88 | <0.001 |  | 0.83 | 0.60-1.15 | 0.267 |
|  |  | multivariate | 0.72 | 0.61-0.84 | <0.001 |  | 0.95 | 0.69-1.31 | 0.755 |
|  | Lp(a) | univariate | 1.00 | 0.91-1.11 | 0.943 |  | 0.97 | 0.80-1.19 | 0.796 |
|  |  | multivariate | 1.02 | 0.92-1.13 | 0.661 |  | 0.95 | 0.79-1.15 | 0.621 |
|  | LDL-C_corr_ | univariate | 0.79 | 0.68-0.91 | <0.001 |  | 0.89 | 0.67-1.18 | 0.415 |
|  |  | multivariate | 0.74 | 0.63-0.86 | <0.001 |  | 0.99 | 0.74-1.32 | 0.930 |
|  | LDL-C_calc_ | univariate | 0.83 | 0.72-0.96 | 0.012 |  | 0.78 | 0.57-1.08 | 0.133 |
|  |  | multivariate | 0.81 | 0.70-0.94 | 0.007 |  | 0.88 | 0.64-1.21 | 0.430 |
|  | HDL-C | univariate | 0.91 | 0.82-1.00 | 0.044 |  | 0.91 | 0.73-1.13 | 0.375 |
|  |  | multivariate | 0.88 | 0.79-0.98 | 0.019 |  | 0.73 | 0.56-0.94 | 0.016 |
|  | Total C | univariate | 0.78 | 0.68-0.89 | <0.001 |  | 0.93 | 0.68-1.25 | 0.620 |
|  |  | multivariate | 0.77 | 0.67-0.89 | <0.001 |  | 1.03 | 0.75-1.40 | 0.874 |
|  | remnant C | univariate | 1.02 | 0.94-1.12 | 0.602 |  | 1.35 | 1.12-1.62 | 0.002 |
|  |  | multivariate | 1.10 | 1.00-1.20 | 0.043 |  | 1.59 | 1.32-1.91 | <0.001 |
|  | triglycerides | univariate | 0.93 | 0.84-1.03 | 0.162 |  | 1.24 | 1.03-1.49 | 0.025 |
|  |  | multivariate | 0.99 | 0.89-1.10 | 0.791 |  | 1.58 | 1.27-1.98 | <0.001 |
|  | apoB-100 | univariate | 0.87 | 0.76-1.00 | 0.052 |  | 1.05 | 0.83-1.33 | 0.667 |
|  |  | multivariate | 0.86 | 0.74-0.99 | 0.041 |  | 1.21 | 0.95-1.52 | 0.117 |
|  | apoA-1 | univariate | 0.85 | 0.77-0.94 | 0.001 |  | 0.94 | 0.75-1.16 | 0.554 |
|  |  | multivariate | 0.83 | 0.74-0.92 | <0.001 |  | 0.78 | 0.62-0.99 | 0.042 |
|  | | | | | | | | | |
| Ceramides and PCs | Cer(d18:1/16:0) | univariate | 1.17 | 1.08-1.27 | <0.001 |  | 1.51 | 1.23-1.83 | <0.001 |
|  |  | multivariate | 1.18 | 1.08-1.29 | <0.001 |  | 1.80 | 1.48-2.20 | <0.001 |
|  | Cer(d18:1/18:0) | univariate | 1.12 | 1.03-1.21 | 0.009 |  | 1.37 | 1.15-1.64 | <0.001 |
|  |  | multivariate | 1.18 | 1.07-1.29 | <0.001 |  | 1.58 | 1.32-1.91 | <0.001 |
|  | Cer(d18:1/24:0) | univariate | 0.75 | 0.67-0.85 | <0.001 |  | 0.95 | 0.79-1.14 | 0.564 |
|  |  | multivariate | 0.82 | 0.72-0.92 | 0.001 |  | 1.17 | 0.96-1.43 | 0.123 |
|  | Cer(d18:1/24:1) | univariate | 1.19 | 1.09-1.31 | <0.001 |  | 1.47 | 1.24-1.73 | <0.001 |
|  |  | multivariate | 1.16 | 1.05-1.28 | 0.003 |  | 1.46 | 1.23-1.72 | <0.001 |
|  | PC 32:0 | univariate | 1.13 | 1.02-1.25 | 0.017 |  | 1.34 | 1.11-1.61 | 0.002 |
|  |  | multivariate | 1.10 | 0.99-1.22 | 0.081 |  | 1.23 | 1.03-1.47 | 0.025 |
|  | PC 36:6 | univariate | 0.68 | 0.61-0.77 | <0.001 |  | 0.71 | 0.58-0.88 | 0.002 |
|  |  | multivariate | 0.73 | 0.65-0.82 | <0.001 |  | 0.80 | 0.64-1.00 | 0.046 |
|  | PC 38:5 | univariate | 0.80 | 0.71-0.89 | <0.001 |  | 1.09 | 0.91-1.31 | 0.346 |
|  |  | multivariate | 0.83 | 0.74-0.93 | 0.001 |  | 1.07 | 0.89-1.28 | 0.467 |
|  | | | | | | | | | |
| Ratios and scores of ceramides and PCs | Cer(d18:1/16:0) / Cer(d18:1/24:0) | univariate | 1.16 | 1.12-1.20 | <0.001 |  | 2.37 | 1.63-3.44 | <0.001 |
|  |  | multivariate | 1.12 | 1.08-1.16 | <0.001 |  | 2.34 | 1.53-3.56 | <0.001 |
|  | Cer(d18:1/18:0) / Cer(d18:1/24:0) | univariate | 1.25 | 1.18-1.32 | <0.001 |  | 1.64 | 1.26-2.14 | <0.001 |
|  |  | multivariate | 1.20 | 1.14-1.27 | <0.001 |  | 1.80 | 1.35-2.41 | <0.001 |
|  | Cer(d18:1/24:1) / Cer(d18:1/24:0) | univariate | 1.15 | 1.11-1.19 | <0.001 |  | 2.68 | 1.90-3-77 | <0.001 |
|  |  | multivariate | 1.11 | 1.07-1.15 | <0.001 |  | 1.90 | 1.35-2.68 | <0.001 |
|  | Cer(d18:1/18:0) / PC 36:6 | univariate | 1.31 | 1.23-1.39 | <0.001 |  | 1.89 | 1.54-2.32 | <0.001 |
|  |  | multivariate | 1.29 | 1.21-1.37 | <0.001 |  | 1.90 | 1.53-2.35 | <0.001 |
|  | Cer(d18:1/24:1) / PC 36:6 | univariate | 1.42 | 1.34-1.51 | <0.001 |  | 2.00 | 1.65-2.43 | <0.001 |
|  |  | multivariate | 1.36 | 1.27-1.45 | <0.001 |  | 2.00 | 1.61-2.49 | <0.001 |
|  |  |  |  |  |  |  |  |  |  |
|  | CERT | univariate | 1.44 | 1.32-1.57 | <0.001 |  | 1.62 | 1.33-1.98 | <0.001 |
|  |  | multivariate | 1.42 | 1.29-1.56 | <0.001 |  | 1.68 | 1.38-2.06 | <0.001 |
|  | CERT2 | univariate | 1.66 | 1.52-1.82 | <0.001 |  | 2.19 | 1.75-2.74 | <0.001 |
|  |  | multivariate | 1.48 | 1.35-1.63 | <0.001 |  | 2.11 | 1.69-2.64 | <0.001 |

*The result of Cox regression analysis are given as Hazard ratios (HR) with the 95% confidence interval (CI) for the low LDL-C (<150mg/dL) subgroups and the high LDL-C (≥150mg/dL) subgroup. HRs were calculated in a univariate and in a multivariate model, after adjustment for age, sex, the status of type 2 diabetes and statin therapy at baseline. HRs are given per 1 Standard Deviation (SD).*

***
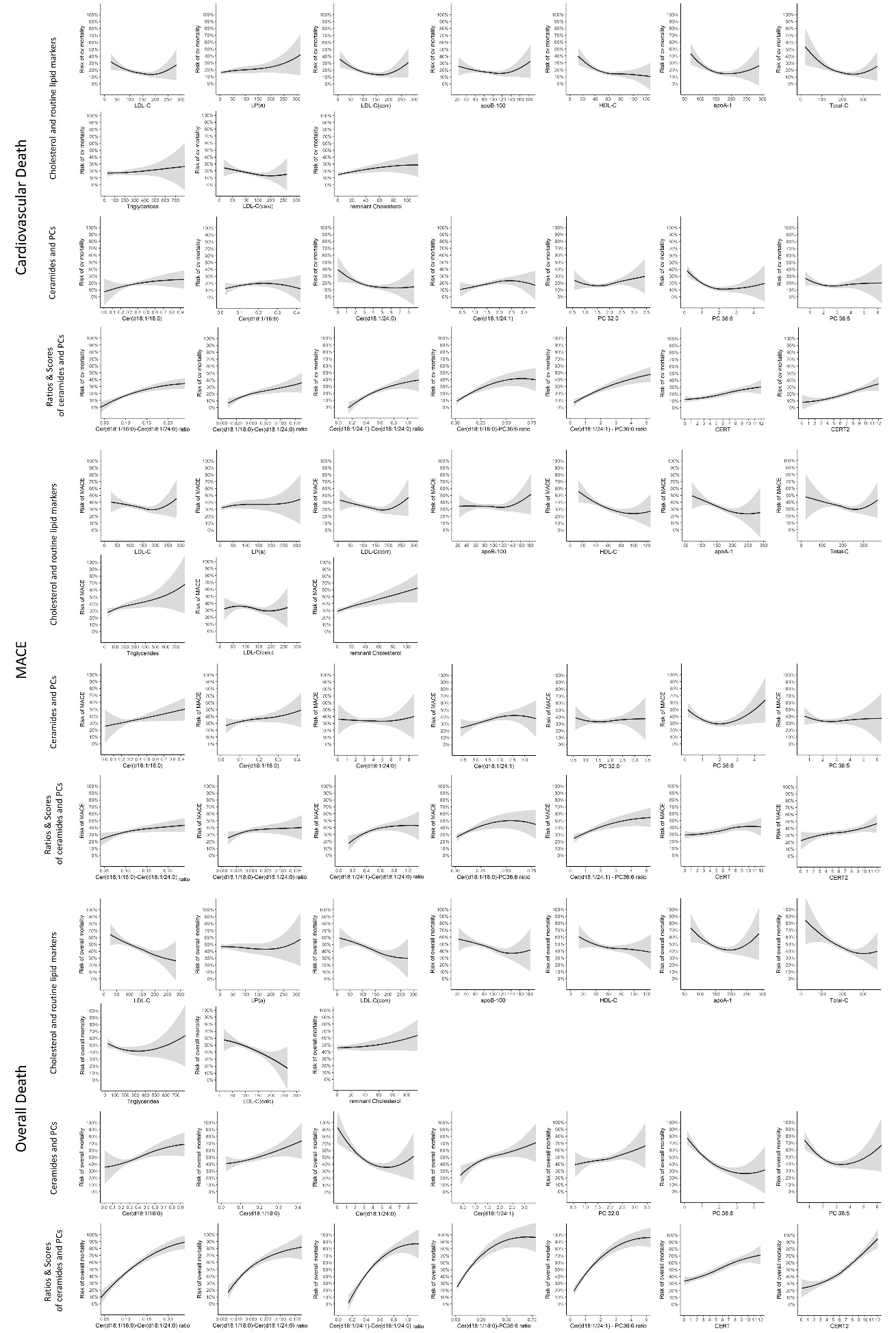
***

***Supplementary figure 1. Risk curves for cardiovascular mortality, MACE, and overall mortality of all lipid markers****. The risk curves are calculated according to loess (LOcally WEighted Scatter-plot Smoother) with 95% confidence intervals (grey). LDL-C, HDL-C, total C, remnant C, LP(a), apoA-1, apoB-100, and triglycerides are given as mg/dL. Ceramide species are given as µmol/L, PC species as peak intensity, and the ratios containing ceramides and PCs as µmol/ (L*peak intensity), and CERT2 is given as a score ranging from 0-12. Data were obtained from single human samples.*

**
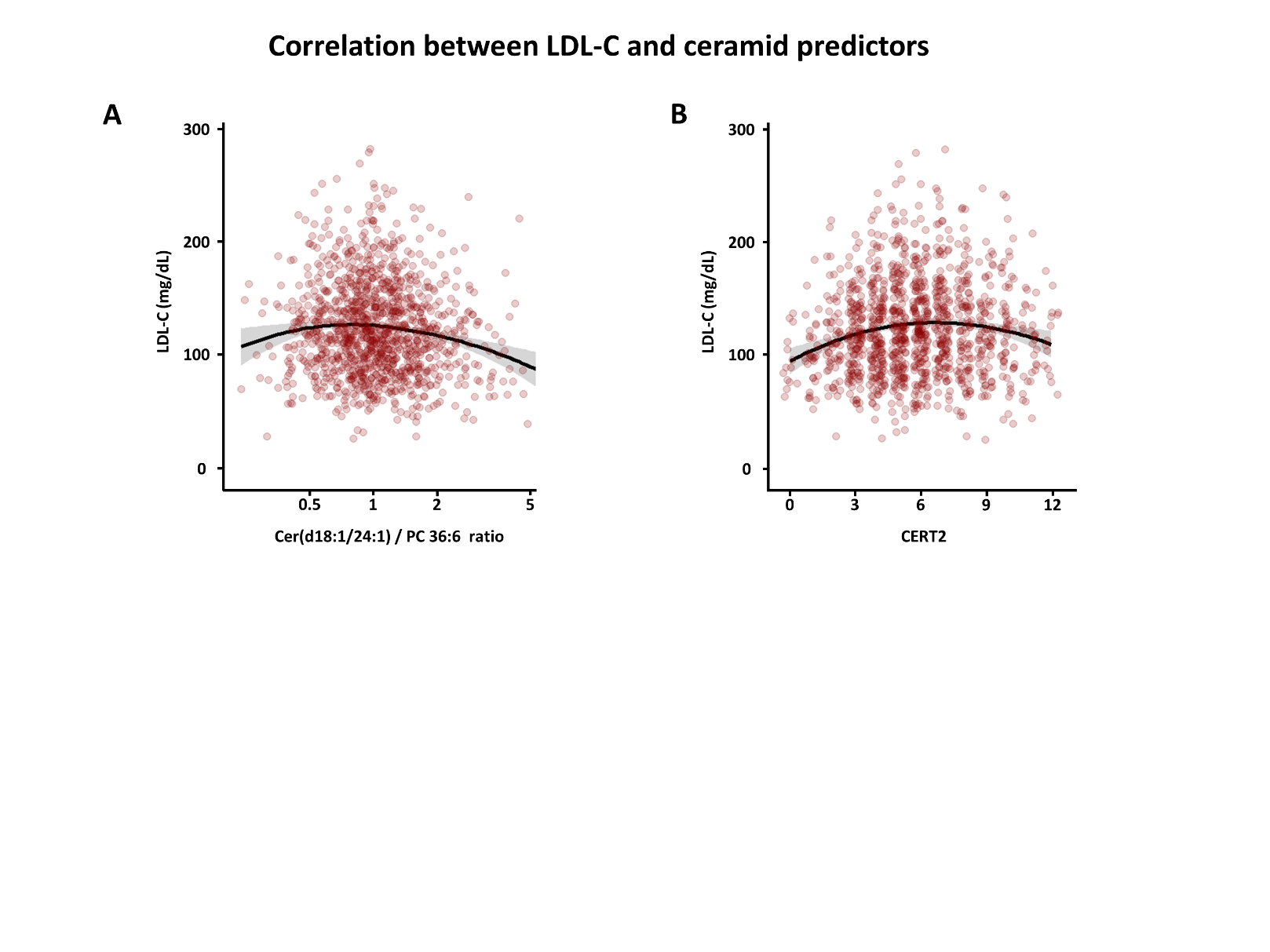
**

***Supplementary figure 2: Correlation between LDL-C and ceramide predictors.*** *The scatter plot demonstrates the relation between LDL-C and the ratio Cer(d18:1/24:1) / PC 36:6 (A) and the relation between LDL-C and CERT2 (B). The curve is calculated by applying loess (black line) with a 95% confidence interval (grey). LDL-C is given as mg/dL, the ratio Cer(d18:1/24:1) / PC 36:6 is given as µmol/ (L*peak intensity), and CERT2 is given as a score ranging from 0-12. Dots in (B) are horizontally jittered. Data were obtained from single human samples.*

*
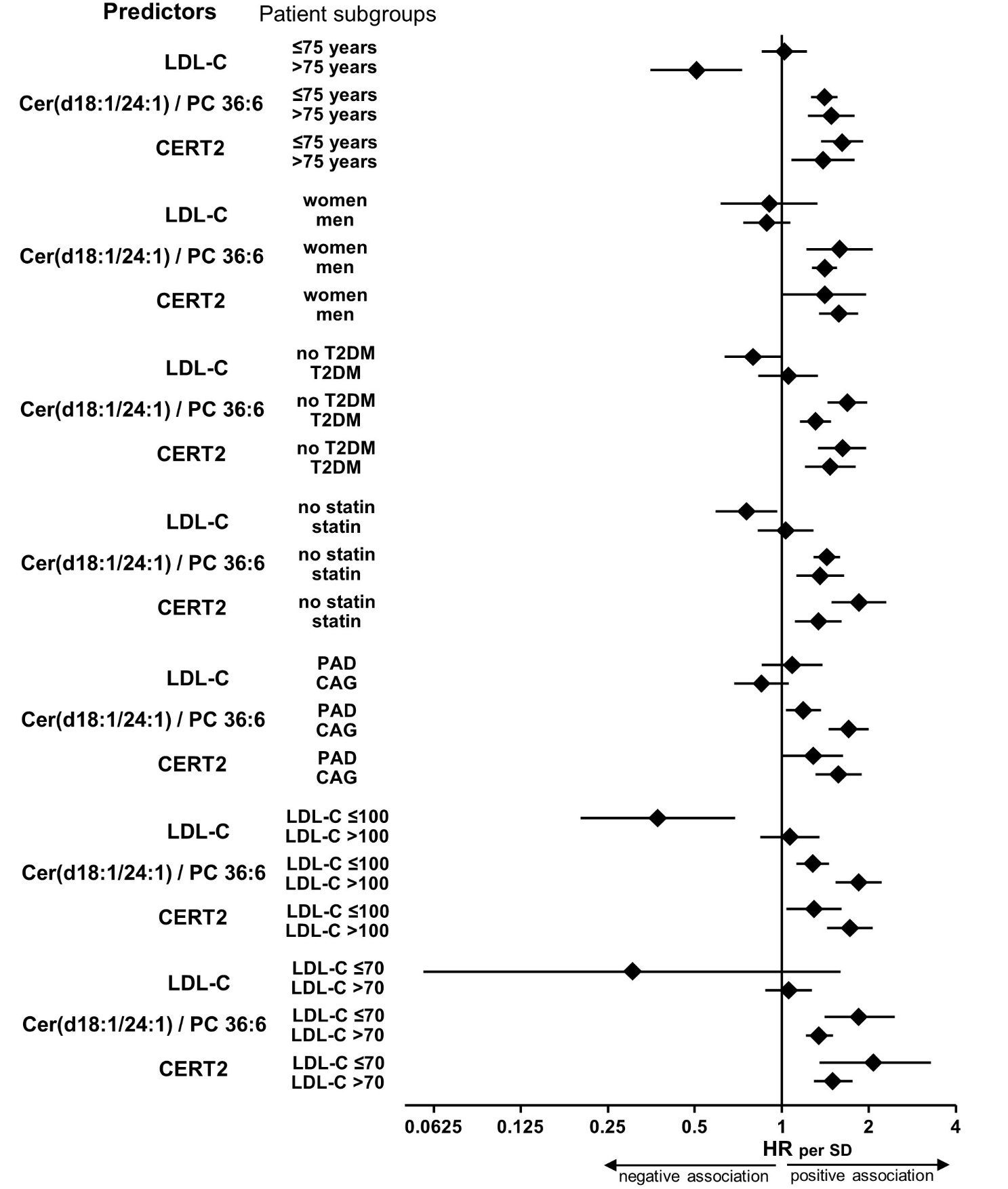
*

***Supplementary figure 3. Association of LDL-C, the ceramide-based ratio Cer(d18:1/24:1) / PC 36:6, and the score CERT2 with the risk of cardiovascular mortality in patient subgroups****. The Forest plots represent the adjusted hazard ratios (HR) of cardiovascular mortality applying a Cox regression model including the covariates age, sex, the status of type 2 diabetes mellitus (T2DM) and the status of statin treatment at baseline as covariates. Patients were stratified regarding their age (≤75 years vs. >75 years), sex, T2DM status, statin treatment status, atherosclerosis manifestation (established PAD vs. referred to CAG), or low / high LDL-C (cut-offs: 100 mg/dL and 70 mg/dL, respectively) at baseline. The HR is given per one SD together with the 95% confidence interval. Data were obtained from single human samples.*
